# Metal-to-insulator crossover in alkali doped zeolite

**DOI:** 10.1038/srep18682

**Published:** 2016-01-04

**Authors:** Mutsuo Igarashi, Peter Jeglič, Andraž Krajnc, Rok Žitko, Takehito Nakano, Yasuo Nozue, Denis Arčon

**Affiliations:** 1 Laboratory for Applied Physics, Department of Electrical Engineering, Gunma College, National Institute of Technology, Toribamachi 580, Maebashi 371-8530, Gunma, Japan; 2 Jožef Stefan Institute, Jamova 39, 1000 Ljubljana, Slovenia; 3 EN-FIST Centre of Excellence, Dunajska 156, 1000 Ljubljana, Slovenia; 4 National Institute of Chemistry, Hajdrihova 19, 1001 Ljubljana, Slovenia; 5 Department of Physics, Graduate School of Science, Osaka University, Toyonaka 560-0043, Osaka, Japan; 6 Faculty of Mathematics and Physics, University of Ljubljana, Jadranska 19, 1000 Ljubljana, Slovenia

## Abstract

We report a systematic nuclear magnetic resonance investigation of the ^23^Na spin-lattice relaxation rate, 1/*T*_1_, in sodium loaded low-silica X (LSX) zeolite, Na_*n*_/Na_12_-LSX, for various loading levels of sodium atoms *n* across the metal-to-insulator crossover. For high loading levels of *n* ≥ 14.2, 1/*T*_1_*T* shows nearly temperature-independent behaviour between 10 K and 25 K consistent with the Korringa relaxation mechanism and the metallic ground state. As the loading levels decrease below *n* ≤ 11.6, the extracted density of states (DOS) at the Fermi level sharply decreases, although a residual DOS at Fermi level is still observed even in the samples that lack the metallic Drude-peak in the optical reflectance. The observed crossover is a result of a complex loading-level dependence of electric potential felt by the electrons confined to zeolite cages, where the electronic correlations and disorder both play an important role.

Zeolites are an important family of materials with periodic arrays of aluminosilicate cages that are widely used in different industrial processes^1^. Moreover, they also show interesting electronic phenomena, when intercalated with alkali metals, associated with the electronic states localized within individual cages. For example, exotic magnetism with different magnetically ordered states has been reported in alkali-doped zeolites^2^,^3^,^4^,^5^,^6^,^7^. With more than 200 possible zeolite frameworks known today^8^, alkali-doped zeolites thus represent a unique playground to control and study the effects of geometry and dopant concentration on the electronic potential depth, electron-electron repulsion and electron-phonon coupling at different length-scales.

Since electronic states are associated to the alkali s-electrons, they remain confined to cages, just like alkali-metals that form clusters or superatoms. It is thus not surprising that the metallic zeolites have been elusive for many years with only one documented exception in rubidium doped zeolite rho, where the microwave conductivity measurements indicated the metallic ground state^9^. Very recently, the insulator-to-metal transition has been also reported in sodium loaded low-silica X (LSX) zeolite, Na_*n*_/Na_12_-LSX^10^,^11^. So far, experimental evidence for the metallic state was mainly limited to the observation of Drude reflection appearing in the infrared region^10^ and a drastic decrease in the resistivity^11^ for the heavily loaded samples. We stress that the measured resistivity is still very high and atypical of simple metals as it does not decrease with decreasing temperature. Additional hint of metallic ground state was provided by a precise x-ray diffraction analysis^12^, where it was shown that Na atoms make bonding network through the tunnel windows that connect zeolite cages and thus establish a precondition for a narrow conduction band. However, firm direct experimental evidence for the metallic state in Na_*n*_/Na_12_-LSX is still lacking.

Nuclear magnetic resonance (NMR) is a powerful local-probe experimental tool to investigate a state of matter even in powder and highly air-sensitive samples. By measuring the temperature dependence of the NMR shift and spin-lattice relaxation time it is in principle possible to distinguish between insulating, metallic and superconducting states^13^,^14^,^15^,^16^. Unfortunately, in alkali-doped zeolites, the spin-lattice relaxation rate, 1/*T*_1_, is dominated by strong fluctuations of local magnetic fields and electric field gradients originating from large amplitude atomic motion of alkali metals^17^,^18^ thus masking the conventional Korringa-like behaviour expected in the metallic state. Here we show for Na_*n*_/Na_12_-LSX that at room temperature the values of ^23^Na 1/*T*_1_ due to the Na motion indeed typically exceed by four orders of magnitude contributions from the coupling of nuclear magnetic moments to itinerant electrons in the metallic state. Cooling sample to cryogenic temperatures freezes out the atomic motions on the NMR time scale and ^23^Na 1/*T*_1_ finally discloses Korringa behaviour below 25 K thus proving the metallic ground state for *n* ≥ 14.2. Surprisingly, a small portion of density of states (DOS) at the Fermi level persists deep into the insulating state. This important finding that was not possible before with bulk-property measurements, holds important clues about the metal-to-insulator crossover in Na_*n*_/Na_12_-LSX, which is here discussed within the correlation-driven and disorder-driven aspects of metal-to-insulator transition (MIT)^19^,^20^.

## Results

**Figure 1:**
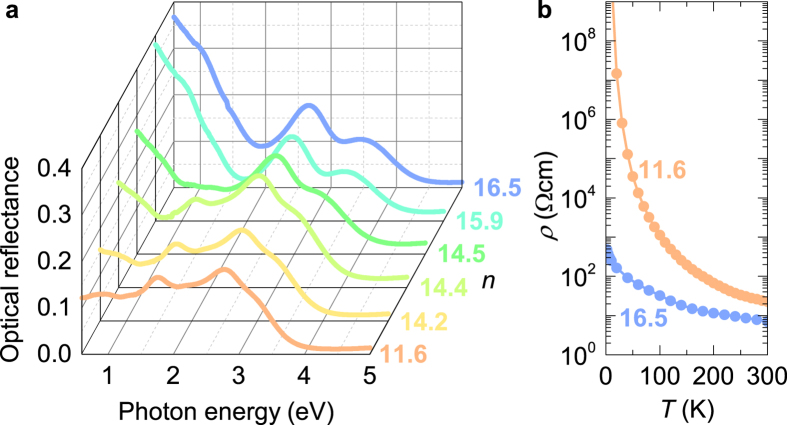
(**a**) Room temperature optical conductivity of Na_*n*_/Na_12_-LSX zeolites as a function of sodium loading level *n*. (**b**) Temperature dependence of resistivity for insulating (*n* = 11.6) and metallic (*n* = 16.5) samples.

The optical reflectance spectra of the Na_*n*_/Na_12_-LSX samples included in this study clearly demonstrate the emergence of Drude peak at lower photon energies for *n* ≥ 14.2 as shown in Fig. 1a. Moreover, the temperature dependence of resistivity reveals finite low-temperature values for *n* = 16.5, but diverges for *n* = 11.6 as displayed in Fig. 1b. The measured resistivity may be affected by the constriction resistance at the connection between powder particles, which can result in a misleading negative temperature coefficient of the resistivity for *n* = 16.5. Nonetheless, finite value of *ρ* at 2 K for *n* = 16.5 demonstrates a finite DOS at the Fermi level, *N*(*E*_*F*_), consistent with the metallic state. These two standard characterization techniques thus comply with the MIT as a function of Na-loading at a critical loading concentration between *n* = 11.6 and *n* = 14.2, in full agreement with the literature data^10^,^11^.

**Figure 2:**
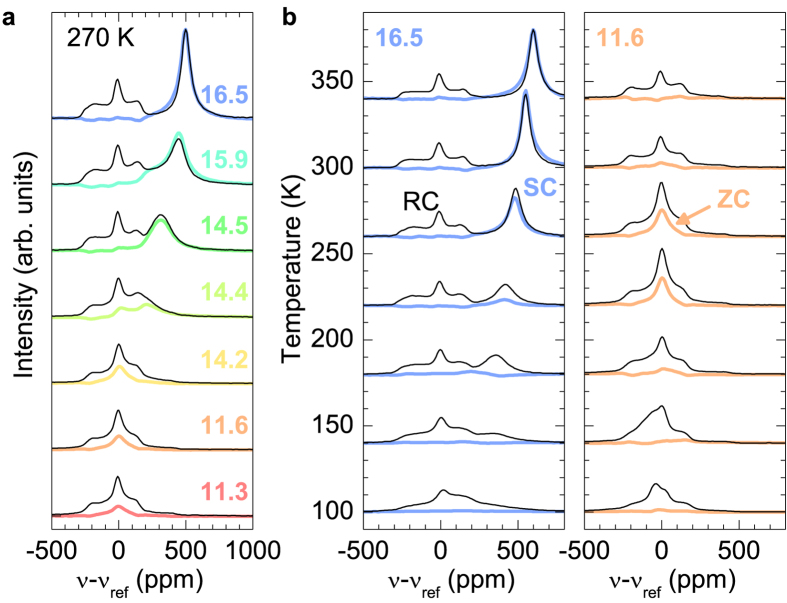
(**a**) ^23^Na NMR spectra at 270 K as a function of loading level *n*, varying between 11.3 and 16.5. (**b**) Temperature dependence of ^23^Na spectra for metallic *n* = 16.5 and insulating *n* = 11.6 samples. All spectra were measured with short (solid black line) and long pulses (thick color line). See text for more details.

At 270 K, the ^23^Na NMR spectrum of insulating Na_11.6_/Na_12_-LSX powder comprises several overlapping peaks close to the Larmor frequency (Fig. 2a). The structure of ^23^Na NMR lineshape reflects the multitude of Na sites in the *β* cages and supercages of the LSX structure^12^,^21^. The contribution of different Na sites to the observed ^23^Na NMR components is discussed in Methods section. The bulk magnetic susceptibility of this sample shows a diamagnetic response, although the presence of diluted localized magnetic moments is evident from a characteristic low-temperature Curie tail^11^. Therefore, the predominately diamagnetic susceptibility of *n* = 11.6 sample suggests that the lineshape and the shift of the ^23^Na NMR spectrum are almost entirely determined by the nuclear chemical shift and quadrupole interactions. The insulating Na_11.6_/Na_12_-LSX sample can be set thus as a suitable NMR reference against which all changes of NMR parameters when crossing the MIT are compared.

Indeed, for samples with *n* ≥ 14.2, a Lorentzian line [hereafter named as a shifted component (SC)] appears in the metallic samples on the high-frequency side of the ^23^Na NMR spectrum, well separated from the diamagnetic frequency range (Fig. 2a). The appearance of SC is limited to samples that show metallic-like response in optical and resistivity measurements (Fig. 1) and is completely absent in insulating samples, e.g. as for *n* = 11.6 shown in Fig. 2b. The SC is optimally detected with an echo pulse sequence with precisely two times longer pulse length than that optimized for the residual diamagnetic ^23^Na spectral component centered around zero shift – hereafter we call it a residual component (RC) as it is reminiscent to that described above for the insulating *n* = 11.6 sample. We conclude that the electric field gradient for Na atoms contributing to this SC is averaged out on the time scale of ^23^Na NMR measurements, ~10^−5^ s. Motional effects also explain the Lorentzian lineshape of SC. EFG averaged to zero and the Lorentzian lineshape are clear signatures that at elevated temperatures Na atoms undergo large amplitude displacements. Temperature dependence of ^23^Na NMR spectra shown for metallic *n* = 16.5 sample in Fig. 2b, reveals that with increasing temperature the intensity of SC increases significantly above ≈150 K. This means that such states must be thermally excited from the ground state. We can rationalize the appearance and the strong temperature dependent shift of SC within a polaron model, where thermally activated behavior is associated with the creation/annihilation of localized small polarons from the bath of (conducting) large polarons^18^.

Unfortunately, the observation of polaronic SC for *n* ≥ 14.2 is only an indirect proof of metallic state. Additional complexity in the analysis of ^23^Na spectra arises for *n* ≤ 14.4, where we observe another non-shifted component (see Fig. 2), which has the optimal pulse lengths the same as SC detected at *n* ≥ 14.2. Using the same arguments as for SC, we conclude that Na atoms contributing to this line must perform large amplitude jumps between different sites in the same cage. However, unlike to SC, this line has no hyperfine interaction with unpaired electron spins, therefore we call it zero component (ZC), implying that it has a completely different, most likely non-magnetic bipolaronic, origin. It is interesting to note that the ^23^Na NMR spectrum for *n* = 14.4 (Fig. 2a) shows a coexistence of ZC and SC, which may indicate an inhomogeneous distribution of sodium atoms throughout the zeolite cages.

**Figure 3:**
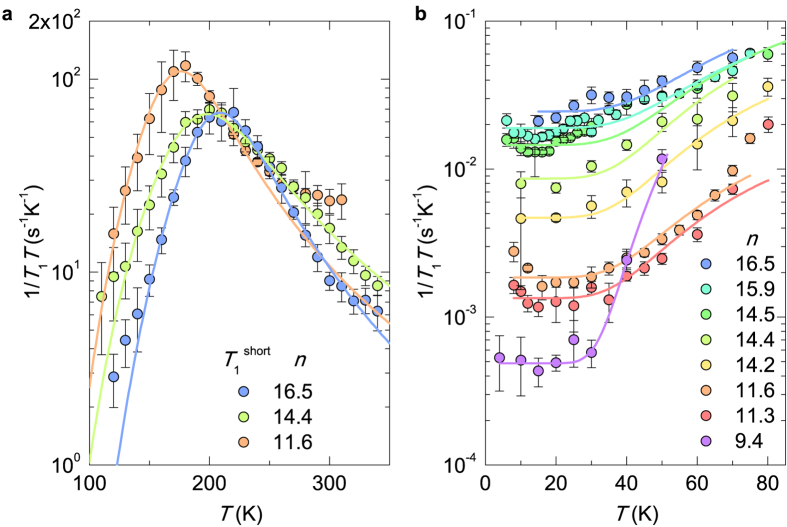
(**a**) Representative temperature dependences of ^23^Na 1/*T*_1_*T* for the SC/ZC component with shorter relaxation time. The solid lines are fits obtained from the BPP model described by Eq. (1). We obtain *E*_*a*_ = (94, 90, 132) meV, *τ*_0_ = (5.8, 15, 1.7) × 10^−12^ s and *C* = (13, 8.8, 9.4) × 10^12^ s^−2^ for *n* = (11.6, 14.4, 16.4), respectively. (**b**) Low-temperature dependences of ^23^Na 1/*T*_1_*T* showing plateau-like behaviour below 25 K for various loading levels *n*. The solids lines are fits obtained by combining the BPP and Korringa relaxation mechanisms [Eq. (3)]. For comparison we added the low-temperature data for the *n* = 9.4 sample, which is according to the optical reflectance and resistivity measurements considered to lie deep in the insulating phase.

Since the metallic state cannot be unambiguously confirmed from the analysis of ^23^Na NMR spectra, we next discuss the spin-lattice relaxation data, where the Korringa behaviour (1/*T*_1_*T* = const.) is usually a signature of metallic state^14^,^22^. For all compositions RC has, compared to ZC and SC, by two orders of magnitude longer *T*_1_. This distinction is manifested over a wide range of temperatures as two separate nuclear magnetization recoveries with long and short time constants. The temperature dependence of the short ZC/SC *T*_1_ component (Fig. 3a), has a pronounced maximum in 1/*T*_1_*T*, which can be between 100 and 350 K empirically modelled within the Bloembergen-Purcell-Pound (BPP)-type mechanism^23^


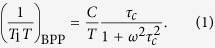


Here *τ*_*c*_ is the correlation time for the local field fluctuations at the nucleus, *ω* is the Larmor angular frequency and *C* is a measure of the fluctuating local fields magnitude. We estimated the activation energy, *E*_*a*_, for the local field fluctuations by assuming the Arrhenius type correlation time, *τ*_*c*_ = *τ*_0_ exp(*E*_*a*_/*k*_*B*_*T*), where *τ*_0_ is a constant. For the activation energies we obtained a value of around 0.1 eV for all loading densities investigated. The estimated activation energy is typical for atomic motion in zeolites^18^ and provides yet another independent proof that sodium motion is present in both insulating and metallic state. However, the strong relaxation due to the atomic motion completely masks the weaker metallic Korringa contribution to the spin-lattice relaxation.

Since *τ*_*c*_ increases exponentially with decreasing temperature, we anticipate that according to Eq. (1) the BPP contribution to the total ^23^Na relaxation rate diminishes at low temperatures, i.e., the atomic-motion driven (1/*T*_1_*T* )_BPP_ → 0 as temperature decreases. In addition, the relaxation rates for RC and ZC/SC become comparable below ~100 K thus implying that other relaxation mechanisms, which are only moderately site dependent, set in. Therefore, the low temperature relaxation curves were fitted with stretched exponential model ~exp[−(*τ*/*T*_1_)^*α*^] with a single effective *T*_1_, where a stretching exponent *α* ≈ 0.6 accounts for distribution of *T*_1_’s due to multiple sites. The most important observation arising from this analysis is that plotting 1/*T*_1_*T* versus temperature for samples with *n* ≥ 14.2, we finally find metallic temperature independent 1/*T*_1_*T* below ~25 K (Fig. 3b). This is a hallmark of the nuclear spin-lattice relaxation via the itinerant electrons and is accounted for by the Korringa expression^14^,^22^


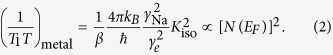


Here *γ*_*e*_ and *γ*_Na_ are the electronic and ^23^Na gyromagnetic ratios, respectively. The phenomenological parameter *β*, called the Korringa factor, characterizes the extent of electron correlations^13^,^14^. The temperature independent isotropic Knight shift, *K*_iso_, which is proportional to Pauli spin susceptibility, is a measure of DOS at the Fermi level, *N*(*E*_*F*_). Comparing the low-temperature 1/*T*_1_*T* values for different *n*, we find that in metallic samples 1/*T*_1_*T* is enhanced relative to insulating samples by an order of magnitude. If the system can be treated as a simple metal and the plateau in 1/*T*_1_*T* is given by the Korringa relaxation mechanism, a monotonic increase of low-temperature 1/*T*_1_*T* with *n* for *n* ≥ 14.2 speaks for a monotonic increase of *N*(*E*_*F*_) with Na loading level. This holds true as long as the relevant electronic bandwidth does not change significantly as a function of loading level. Because these compounds are far away from any magnetic instability, we expect that the electron correlations could be well taken into account by the Korringa factor. As a result, the electron correlation can only renormalize the extracted values of *N*(*E*_*F*_) and could thus hardly affect the deduced monotonic increase in *N*(*E*_*F*_) as a function of loading level.

In order to quantitatively extract (1/*T*_1_*T* )_metal_ from the measured temperature dependence of 1/*T*_1_*T*, we assumed that the relaxation rate has two contributions, the BPP and Korringa, described by Eqs. (1) and (2), respectively. That is, the data was fitted to


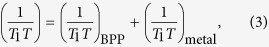


where between 30 K and 80 K another sodium motion likely sets in with an estimated activation energy of around 22 meV for all sodium loading densities investigated (except for *n* = 9.4, where we obtained a slightly higher value of 32 meV). Table 1 summarizes the values of (1/*T*_1_*T* )_metal_ for all *n* in the range between 9.4 and 16.5 and is compared to the related value measured in bulk metallic Na. We note that the (1/*T*_1_*T* )_metal_ values are by more than one order of magnitude smaller than that of bulk metallic Na thus indicating a relatively low *N*(*E*_*F*_) in these metallic samples. This conclusion is further supported, if we use (1/*T*_1_*T* )_metal_ = 2.3 × 10^−2^ s^−1^ K^−1^ of *n* = 16.5 sample to calculate *K*_iso_ = 300 ppm from Eq. (2) and compare it to much larger value of 1120 ppm found in metallic sodium^14^. Surprisingly, the plateau-like low-temperature behaviour in 1/*T*_1_*T* is also seen for *n* = 11.6 and 11.3, where the Drude term is not observed and resistivity diverges at low temperature. We stress that the observed low-temperature 1/*T*_1_*T* clearly rules out the possibility that for this loading range the system can be discussed as a narrow-gap semiconductor. In this case, 1/*T*_1_*T* would be dominated by the exp(−Δ/*k*_*B*_*T* ) term for 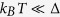, which decays exponentially with decreasing temperature^24^, in disagreement with the experimental data.

## Discussion

**Figure 4:**
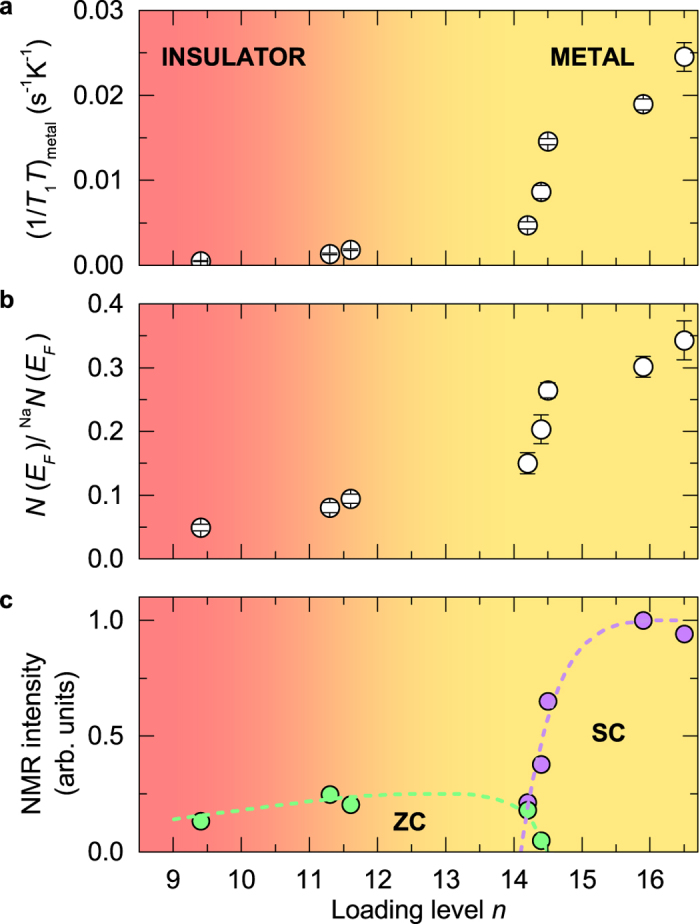
The phase diagram of Na_*n*_/Na_12_-LSX zeolite showing the values of (**a**) (1/*T*_1_*T* )_metal_, (**b**) normalized *N*(*E*_*F*_) and (**c**) NMR spectrum intensity for ZC and SC as a function of Na loading level *n*. The colour gradient divides the insulating and metallic regions as experimentally observed from the resistivity and optical conductivity measurements.

Plotting the normalized *N*(*E*_*F*_) extracted from Eq. (2) versus the sodium loading level *n* (Fig. 4b), we find that *N*(*E*_*F*_) markedly increases with *n* for *n* ≥ 14.2, thus speaking for the enhancement of DOS at Fermi level *N*(*E*_*F*_) with doping, which is in qualitative agreement with the enhancement of optical reflectance (Fig. 1a). We note that for the most loaded sample (*n* = 16.5), the extracted *N*(*E*_*F*_) is by a factor of ~3 smaller than the corresponding value in bulk Na. Our study of a minimal single-orbital Hubbard model demonstrated^25^ that the experimentally observed strong variation of Drude peak in optical conductivity cannot be explained solely by band filling effects. In fact, it showed that the variation of electron-electron repulsion *U* divided by a bandwidth *W*, is much more relevant, meaning that the main effect of sodium loading is to define the electronic potential and the Coulomb repulsion felt by the electrons in the zeolite cages. Similarly, the importance of electron correlations has been theoretically^26^ and experimentally^27^,^28^ recognized for related potassium-loaded LTA and FAU zeolites. However, the correlation-driven MIT is expected to be of first order^19^, which is not directly supported by the present data. We recall that a finite *N*(*E*_*F*_) has been observed even in the nominally insulating samples of Na_*n*_/Na_12_-LSX. For example, the *n* = 11.3 sample exhibits a resistivity diverging at low temperatures and is described by an energy band gap of 0.2 eV. At the same time, the local probe ^23^Na NMR for this sample shows the finite *N*(*E*_*F*_), more precisely ~8% of the value found in metallic sodium. The phase diagram shown in Fig. 4 is more reminiscent of a metal-to-insulator crossover rather than the sharp transition thus calling for considerations of other relevant microscopic factors.

In the alternative picture, where disorder is the driving mechanism for MIT, a continuous metal-to-insulator is typically found at finite temperatures^29^. The physical reason is that at finite temperatures the electrons can escape the trapping potential through the thermal activation. Indeed, the alkali-doped zeolites can be viewed as a strongly disordered system for several reasons. First, by a careful analysis of the spectral intensities belonging to SC and ZC as a function of sodium loading level (Fig. 4c), we identify a region around *n* = 14.4 where both spectral components coexist, implying an inhomogeneous Na distribution in the cages that is responsible for a cage-to-cage variation in the electric potential depths. Second, as pointed out in the x-ray study by Ikeda *et al.*^12^, in Na_*n*_/Na_12_-LSX the coordinates of available sodium sites in the zeolite framework remain the same as a function of sodium loading level. What is changing with *n* is the average occupancy of these sites. Local variations in the Na arrangements in the neighbouring cages are responsible for the variations in the local potential depth and thus for the disorder. For low carrier densities, the trapping potential of disordered sodium clusters within the cages is expected to become comparable or larger than the Fermi level, and the electrons get localized. At higher loading densities not only the carrier density increases, but since the loaded sodium atoms reach the limit of the highest possible occupancy in the cages^12^, the disorder strength decreases and triggers the MIT.

However, at the critical loading densities yet another possibility of a percolation-type metal-to-insulator transition^19^ opens. Although, the disorder-driven scenario in the presence of varying correlation effects seems plausible, we should be aware that the variation of alkali atom loading level not only changes the electron correlation energies and disorder strength, but also strongly determines the electric potential felt by the electrons. Electron-density distribution analysis showed a formation of the chain-like Na cation distribution in metallic Na_16.7_/Na_12_-LSX, which connects the neighboring supercages^12^. This Na-Na connectivity is not formed in nominally insulating Na_9.4_/Na_12_-LSX. In the percolation picture of a random (disordered) potential a small metallic regions of connected supercages are formed at low sodium loading densities separated by insulating areas. When the electron density increases the metallic regions grow and eventually become connected at the percolation threshold^19^. Although our observation of the crossover regime and the inhomogeneous distribution of sodium atoms throughout the zeolite lattice tentatively support the percolation picture, we leave the important question of the true nature of MIT in alkali doped zeolites open for further studies. However, what is made clear from present results is that for the Na_*n*_/Na_12_-LSX zeolites the concentration of sodium atoms strongly affects in a very complex way the electronic properties by simultaneously changing the band filling, the electronic potential depth, the electron-electron repulsion and the amount of disorder.

## Conclusions

Using NMR as a local probe of sodium loaded low-silica X zeolite (Na_*n*_/Na_12_-LSX), we have unambiguously confirmed a metallic ground state for higher loading densities of *n* ≥ 14.2. By extracting the DOS at the Fermi level as a function of sodium loading level, we have discovered a continuous (crossover like) evolution across the metal-to-insulator transition. Most importantly, a finite DOS at the Fermi level for nominally insulating samples and a clear indication of inhomogeneous Na distribution in the neighboring cages in the crossover region, put some constraints on the driving mechanism of electron localization and the nature of MIT in alkali-doped zeolites.

## Methods

### Samples

The LSX zeolites have a chemical formula A_12_Al_12_Si_12_O_48_, where A stands for alkali-metal cations that are required for charge compensation of the aluminosilicate framework^1^. The main structural motif is comprised of truncated octahedral *β* cages, which are arranged in a diamond structure by doubly connecting their 6-membered rings. This way additional supercages are formed with a diameter approximately twice of that of *β* cages. Following the Lowenstein’s rule^30^ the Si and Al atoms alternatingly occupy the framework sites resulting in structurally ordered LSX zeolite framework. When sodium-based parent structures, i.e. A = Na, hereafter abbreviated as Na_12_-LSX, are exposed to Na vapour following the standard procedure described elsewhere^11^, a controlled amount of Na is additionally loaded yielding a targeted composition Na_*n*_/Na_12_-LSX. Here *n* denotes the loading density of guest Na atoms per supercage. The values of *n* were for the purpose of this study recalibrated by inductively coupled plasma technique. Particularly for higher density levels, the calibrated values substantially exceed those calculated from known amount of starting materials used in our preliminary NMR study^18^. For example, the loading levels of “*n* = 10” and “*n* = 12” studied in ref. 18 correspond to the recalibrated values of *n* = 9.4 and *n* = 16.7, respectively.

### Bulk-property measurements

Optical reflectance spectra were measured by conventional apparatus. Since we were dealing with powder samples with a grain diameter of few *μ*m, the resistivity, *ρ*, was measured by pinching the powder between two metallic plates acting also as terminals^11^.

### NMR measurements

Here we present detailed ^23^Na NMR experiments on Na_*n*_/Na_12_-LSX samples in the Na-loading range 9.4 ≤ *n* ≤ 16.5. The ^23^Na (*I* = 3/2) NMR spectra and *T*_1_ were measured in a magnetic field of 4.7 T in the temperature range between 6 K and 340 K. The ^23^Na reference frequencies of 52.9055 MHz was determined from NaCl aqueous solution standards.

### ^23^Na NMR signal assignment to the sodium crystallographic sites

According to ref. 12, the occupancies of Na1, Na2, Na3 and Na6 crystallographic sites remain the same for *n* = 9.4 and *n* = 16.7. These sites are located near the inner wall of the zeolite framework and are responsible for the framework charge compensation. They can be naturally attributed to the RC component present at all sodium loading levels. On other hand, the occupancies of Na5, Na7, Na8 and Na9 sites, which are located near the centre of the supercage, increase substantially from *n* = 9.4 to *n* = 16.7. Based on this observation we tentatively attribute the SC to this set of sites. Next we recall that the ZC and SC behave very similarly since both signals are influenced by sodium motion and the only difference is the absence of NMR shift for ZC. As proposed in ref. 18, the SC detects small polarons excited from the bath of large polarons, whereas the ZC could be explained by the non-magnetic bipolaronic nature of electronic states at lower loading levels.

## Additional Information

**How to cite this article**: Igarashi, M. *et al.* Metal-to-insulator crossover in alkali doped zeolite. *Sci. Rep.* **6**, 18682; doi: 10.1038/srep18682 (2016).

## Figures and Tables

**Table 1:** Extracted values of ^23^Na (1/*T*_1_*T* )_metal_ as a function of Na loading level *n*.

Loading level *n*	(1/*T*_1_*T*)_metal_ (s^−1^ K^−1^)	*N*(*E*_*F*_)/^Na^*N*(*E*_*F*_)
16.5	0.0245 ± 0.0017	0.343 ± 0.031
15.9	0.0190 ± 0.0006	0.302 ± 0.016
14.5	0.0146 ± 0.0004	0.264 ± 0.012
14.4	0.0086 ± 0.0008	0.204 ± 0.023
14.2	0.0047 ± 0.0004	0.150 ± 0.017
11.6	0.0019 ± 0.0001	0.094 ± 0.007
11.3	0.0013 ± 0.0001	0.080 ± 0.008
9.4	0.0005 ± 0.0001	0.049 ± 0.005

The values of *N*(*E*_*F*_) normalized to metallic sodium ^Na^*N*(*E*_*F*_) listed in column three are calculated using Eq. (2) and taking (1/*T*_1_*T* ) = 0.210 ± 0.004 s^−1^ K^−1^ of metallic sodium^14^.

## References

[b1] Breck D. W. Zeolite molecular sieves: structure, chemistry, and use (Wiley, New York, 1973).

[b2] Nozue Y., Kodaira T. & Goto T. Ferromagnetism of potassium clusters incorporated into zeolite LTA. Phys. Rev. Lett. 68, 3789 (1992). 10045797 10.1103/PhysRevLett.68.3789

[b3] Srdanov V. I., Stucky G. D., Lippmaa E. & Engelhardt G. Evidence for an antiferromagnetic transition in a zeolite-supported cubic lattice of F centers. Phys. Rev. Lett. 80, 2449 (1998).

[b4] Damjanovic L., Stucky G. D. & Srdanov V. I. Magnetism of F centers; indication of an antiferromagnetic phase transition in potassium-electro-sodalite. J. Serb. Chem. Soc. 65, 311 (2000).

[b5] Nakano T. *et al.* *μ*SR study on ferrimagnetic properties of potassium clusters incorporated into low silica X zeolite. Physica B 374–375, 21 (2006).

[b6] Nakano T., Matsuura M., Hanazawa A., Hirota K. & Nozue Y. Direct observation by neutron diffraction of antiferromagnetic ordering in *s* electrons confined in regular nanospace of sodalite. Phys. Rev. Lett. 109, 167208 (2012). 23215125 10.1103/PhysRevLett.109.167208

[b7] Nakano T. *et al.* Exotic magnetism of *s*-electron cluster arrays: ferromagnetism, ferrimagnetism and antiferromagnetism. J. Korean Phys. Soc. 63, 699 (2013).

[b8] Verheyen E. *et al.* Design of zeolite by inverse sigma transformation. Nature Mater. 11, 1059 (2012). 23085567 10.1038/nmat3455

[b9] Anderson P. A. *et al.* Rubidium doped zeolite rho: structure and microwave conductivity of a metallic zeolite. Dalton Trans. 3122 (2004). 15452642 10.1039/b402668c

[b10] Nakano T., Mizukane T. & Nozue Y. Insulating state of Na clusters and their metallic transition in low-silica X zeolite. J. Phys. Chem. Solids 71, 650 (2010).

[b11] Nozue Y., Amako Y., Kawano R., Mizukane T. & Nakano T. Insulating state and metallic phase transition of heavily sodium-doped low-silica X (LSX) zeolites. J. Phys. Chem. Solids 73, 1538 (2012).

[b12] Ikeda T., Nakano T. & Nozue Y. Crystal structures of heavily Na-loaded low-silica X (LSX) zeolites in insulating and metallic states. J. Phys. Chem. C 118, 23202 (2014).

[b13] Pennington C. H. & Stenger V. A. Nuclear magnetic resonance of C_60_ and fulleride superconductors. Rev. Mod. Phys. 68, 855 (1996).

[b14] Walstedt R. E. The NMR Probe of High-T_c_ Materials (Springer Verlag, Berlin, 2008).

[b15] Grafe H.-J. *et al.* ^75^As NMR studies of superconducting LaFeAsO_0.9_F_0.1_. Phys. Rev. Lett. 101, 047003 (2008). 18764358 10.1103/PhysRevLett.101.047003

[b16] Potočnik A. *et al.* Size and symmetry of the superconducting gap in the f.c.c. Cs_3_C_60_ polymorph close to the metal-Mott insulator boundary. Sci. Rep. 4, 4265 (2014). 24584087 10.1038/srep04265 PMC3939459

[b17] Heinmaa I., Vija S. & Lippmaa E. NMR study of antiferromagnetic black sodalite Na_8_(AlSiO_4_)_6_. Chem. Phys. Lett. 327, 131 (2000).

[b18] Igarashi M. *et al.* NMR study of thermally activated paramagnetism in metallic low-silica X zeolite filled with sodium atoms. Phys. Rev. B 87, 075138 (2013).

[b19] Dobrosavljević V. Introduction to metal-insulator transitions. In Dobrosavljević V., Trivedi N. & Valles J. M. Jr. (eds.) Conductor Insulator Quantum Phase Transitions (Oxford University Press, Oxford, 2012).

[b20] Siegrist T. *et al.* Disorder-induced localization in crystalline phase-change materials. Nature Mater. 10, 202 (2011). 21217692 10.1038/nmat2934

[b21] Feuerstein M., Engelhardt G., McDaniel P. L., MacDougall J. E. & Gaffney T. R. Solid-state nuclear magnetic resonance investigation of cation siting in LiNaLSX zeolites. Microporous Mesoporous Mater. 26, 27 (1998).

[b22] Slichter C. P. Principles of Magnetic Resonance (Springer-Verlag, Berlin, 1989).

[b23] Bloembergen N., Purcell E. M. & Pound R. V. Relaxation effects in nuclear magnetic resonance absorption. Phys. Rev. 73, 679 (1948).

[b24] Grykałowska A. & Nowak B. Nuclear spin-lattice relaxation in narrow gap semiconductors TiPtSn and ZrPtSn. Intermetallics 15, 1479 (2007).

[b25] Žitko R., Osolin Ž. & Jeglič P. Repulsive versus attractive Hubbard model: Transport properties and spin-lattice relaxation rate. Phys. Rev. B 91, 155111 (2015).

[b26] Arita R. *et al.* Electronic properties of alkali-metal loaded zeolites: Supercrystal Mott insulators. Phys. Rev. B 69, 195106 (2004).

[b27] Nakano T., Ikemoto Y. & Nozue Y. Ferromagnetism and paramagnetism in potassium clusters incorporated in zeolite LTA. Eur. Phys. J. D 9, 505 (1999).

[b28] Ikemoto Y., Nakano T., Kuno M. & Nozue Y. Magnetic and optical properties of K and Na clusters arrayed in a diamond structure in zeolite FAU. Physics B 281&282, 691 (2000).

[b29] Rosenbaum T. F., Andres K., Thomas G. A. & Bhatt R. N. Sharp metal-insulator transition in a random solid. Phys. Rev. Lett. 45, 1723 (1980).

[b30] Lowenstein W. The distribution of aluminum in the tetrahedra of silicates and aluminates. Am. Mineral. 39, 92 (1954).

